# Melatonin Treatments Reduce Chilling Injury and Delay Ripening, Leading to Maintenance of Quality in Cherimoya Fruit

**DOI:** 10.3390/ijms24043787

**Published:** 2023-02-14

**Authors:** Jorge Medina-Santamarina, Fabián Guillén, Mihaela Iasmina Madalina Ilea, María Celeste Ruiz-Aracil, Daniel Valero, Salvador Castillo, María Serrano

**Affiliations:** 1 Department of Food Technology-CIAGRO, University Miguel Hernández, Ctra. Beniel, km 3.2, 03312 Orihuela, Alicante, Spain; 2 Department of Applied Biology-CIAGRO, University Miguel Hernández, Ctra. Beniel, km 3.2, 03312 Orihuela, Alicante, Spain

**Keywords:** *Annona cherimola*, ion leakage, chlorophyll, firmness, total soluble solids, titratable acidity, ethylene

## Abstract

Spain is the world’s leading producer of cherimoya, a climacteric fruit highly appreciated by consumers. However, this fruit species is very sensitive to chilling injury (CI), which limits its storage. In the present experiments, the effects of melatonin applied as dipping treatment on cherimoya fruit CI, postharvest ripening and quality properties were evaluated during storage at 7 °C + 2 days at 20 °C. The results showed that melatonin treatments (0.01, 0.05, 0.1 mM) delayed CI, ion leakage, chlorophyll losses and the increases in total phenolic content and hydrophilic and lipophilic antioxidant activities in cherimoya peel for 2 weeks with respect to controls. In addition, the increases in total soluble solids and titratable acidity in flesh tissue were also delayed in melatonin-treated fruit, and there was also reduced firmness loss compared with the control, the highest effects being found for the 0.05 mM dose. This treatment led to maintenance of fruit quality traits and to increases in the storage time up to 21 days, 14 days more than the control fruit. Thus, melatonin treatment, especially at 0.05 mM concentration, could be a useful tool to decrease CI damage in cherimoya fruit, with additional effects on retarding postharvest ripening and senescence processes and on maintaining quality parameters. These effects were attributed to a delay in the climacteric ethylene production, which was delayed for 1, 2 and 3 weeks for 0.01, 0.1 and 0.05 mM doses, respectively. However, the effects of melatonin on gene expression and the activity of the enzymes involved in ethylene production deserves further research.

## 1. Introduction

Cherimoya (*Annona cherimola* Mill), also named chirimoya, is an edible fruit tree belonging to the family Annonaceae, and although its origin is still under discussion, it is considered that it is native from the Mesoamerican region, being already cultivated in 1200 BCE in the Inca empire [1,2]. The cherimoya was introduced from America to Spain between the 16th and 18th centuries, with the primary available record in 1757, and then it was distributed to tropical and subtropical areas of Europe, Africa and Asia. The main worldwide producer of cherimoya fruit is Spain, but also Peru and Chile are important cherimoya producers, and the most widely spread cultivar around the world is ‘Fino de Jete’.

Cherimoya fruit is highly appreciated by consumers due to this excellent taste and flavour, and also because of its phytochemical content with health beneficial effects, such as phenolics and vitamins C and E as well as its high concentration in essential minerals, mainly K, Ca, Fe, and Zn [2,3,4,5]. However, the quality parameters of cherimoya fruit change quickly after harvest due to its fast climacteric ripening process [6,7], leading to rapid quality losses and a short shelf life, which limits its market potential. Cold storage is the most used technique to preserve fruit quality, but cherimoya, as other tropical and subtropical fruit species, is susceptible to chilling injury (CI), with symptoms such as abnormal ripening and browning [8,9]. The safe temperatures for prolonged cold storage of cherimoya without affecting organoleptic properties and avoiding development of chilling injury-related symptoms range from 8 to 15 °C depending on the cultivar [7,8,10].

Thus, there have been various attempts to avoid CI during cherimoya storage at low temperature in order to increase its storage period while maintaining its high quality attributes. In this sense, Alique and Oliveira [11] showed that the combination of 3 and 6 KPa CO_2_ with 3 KPa O_2_ had an additive effect on reducing CI and extended the maintenance of quality properties in ‘Fino de Jete’ cherimoya during storage at 9 °C for two weeks with respect to fruit stored in air. CI was also reduced in this cherimoya cultivar by a treatment with 20% CO_2_ for 3 days before storage at 6 °C [12]. Accordingly, Tinebra et al. [13] have recently reported that modified atmosphere packaging (MAP with 21% O_2_ and 1% CO_2_) and active-MAP (10% O_2_ and 30% CO_2_) maintained quality of cherimoya during storage at 10 °C compared with control fruit stored in open air. Heat treatment at 55 °C for 5 h was also reported to alleviate CI symptoms when cherimoya fruit were stored at 4 °C, due to the synthesis of small molecular size heat-shock proteins (sHSPs) involved in preventing and/or repairing stress induced damage [14].

Melatonin is an endogenous indole compound with multiple biological functions in plants [15,16,17] from seed germination to stress tolerance and fruit growth and ripening. It is synthesised from tryptophan, which is converted into melatonin in a two-step pathway. Then, serotonin is converted into melatonin through two different pathways involving the intermediates N-acetyl serotonin and 5-methoxy-tryptamine [18]. Melatonin has been reported to reduce CI in a wide range of fruit species when applied as a postharvest treatment, with additional effects on delaying ripening and senescence processes, leading to maintenance of fruit quality traits [19,20,21]. For instance, CI symptoms were delayed by 1 and 2.5 mM melatonin dipping treatment for 15 min in ‘Friar’ plum [22], and delays were also achieved with 0.1 mM melatonin dipping for 2 h in several mango cultivars [23]. Accordingly, CI symptoms, manifested as flesh browning and translucency, were reduced in pineapple after 21 days of storage at 9 °C followed by 4 days at 22 °C as a consequence of 0.1 mM melatonin dipping treatment for 10 min [24]. Melatonin treatment has been also reported to be effective in increasing zucchini fruit tolerance to CI, although this effect was enhanced when melatonin and 1-methylcyclopropene treatments were combined [25]. In addition, melatonin has been reported to extend the shelf life of fruit when stored at non-chilling temperatures in a wide range of fruit species, including mango, banana and pear, among other [19,20,21,26,27,28]

Based on these previous reports, it was hypothesised that postharvest melatonin treatment could reduce CI in cherimoya fruit and delay the postharvest ripening process leading to maintenance of fruit quality traits during storage. Thus, the main goal of the present research was to increase CI tolerance of cherimoya fruit by melatonin treatment, which is a new approach since as far as we know, no previous reports about this issue are available in the literature. The effects of melatonin treatments on fruit quality attributes and ripening process were also analysed.

## 2. Results

### 2.1. Experiment 1—Selection of the Most Appropriate Melatonin Treatment

Melatonin treatments, applied at 0.1, 0.3, 0.5 and 1 mM as dipping for 10, 60 and 180 min, reduced weight losses after 14 days of storage at 7 °C + 2 days at 20 °C, although significant (*p* < 0.05) differences were observed among melatonin concentrations and dipping times. The lowest weight losses were observed for 10 min of dipping treatments for all the melatonin concentrations tested, and a 0.1 mM concentration was the most effective dose (Figure S1). Fruit firmness at harvest was 36.45 ± 2.42 N mm^−1^ and decreased sharply during storage, reaching values lower than 10 N mm^−1^ in control fruit after 14 days of cold storage + 2 days at 20 °C. However, melatonin treatments reduced significantly (*p* < 0.05) the softening process, and the highest firmness levels were found in 0.1 mM melatonin dipping treatment for 10 min (Figure S2). Thus, 0.1 mM melatonin concentration and dipping treatment for 10 min was selected for the next experiment, in which two additional melatonin concentrations were assayed, 0.05 and 0.01 mM.

### 2.2. Effects of Melatonin Treatment on Cherimoya CI, Quality Parameters and Ripening

#### 2.2.1. Chilling Injury and External Quality Parameters

**Figure 1:**
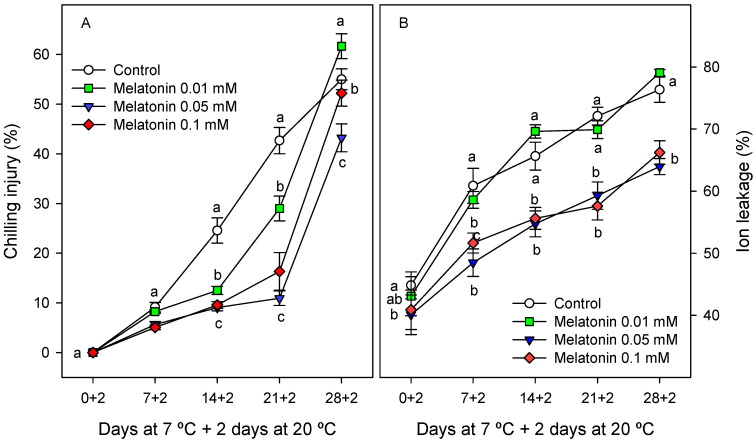
Chilling injury (**A**) and ion leakage (**B**) in cherimoya peel as affected by melatonin (0.01, 0.05 and 0.1 mM) treatments. Data are the mean ± SE of three replicates. Different letters show significant differences (*p* < 0.05) among treatments for each sampling date.

Chilling injury (CI) symptoms, manifested as peel browning, increased during storage, although melatonin-treated fruit showed significantly (*p* < 0.05) lower CI symptoms than controls from the first week of storage, especially with 0.1 and 0.05 mM doses (Figure 1A). A similar trend was observed for ion leakage (IL), that is to say, it increased with storage with significant (*p* < 0.05) higher values in the control and 0.01 mM melatonin-treated fruit than in the 0.05- and 0.1 mM-treated fruit (Figure 1B).

**Figure 2:**
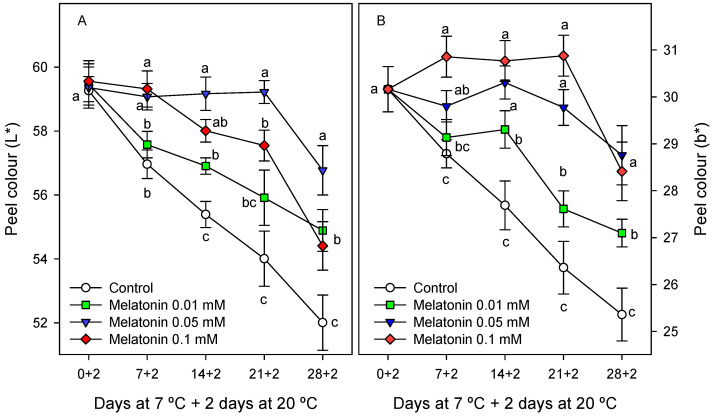
L* (**A**) and b* (**B**) colour parameters in cherimoya peel as affected by melatonin (0.01, 0.05 and 0.1 mM) treatments. Data are the mean ± SE of three replicates. Different letters show significant differences (*p* < 0.05) among treatments for each sapling date.

**Figure 3:**
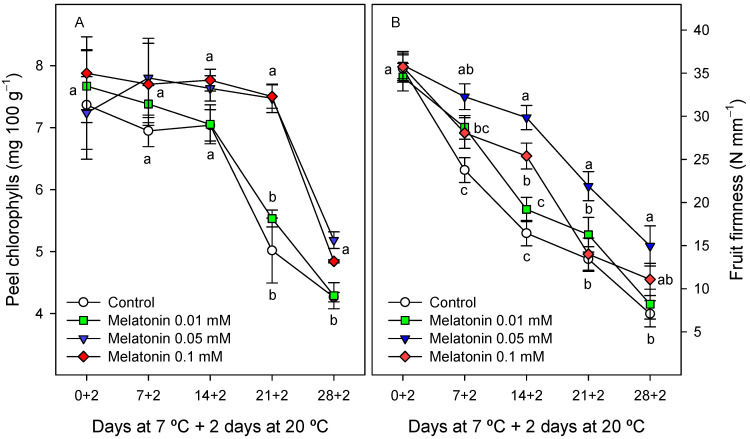
Peel chlorophyll concentration (**A**) and fruit firmness (**B**) as affected by melatonin (0.01, 0.05 and 0.1 mM) treatments. Data are the mean ± SE of three replicates. Different letters show significant differences (*p* < 0.05) among treatments for each sapling date.

Significant differences in peel colour, measured as CIElab coordinates, were also found between the control and melatonin-treated fruit, the highest being observed for the L* and b* parameters, which decreased during storage from the first sampling date in control fruit, whereas these changes were significantly (*p* < 0.05) delayed in melatonin-treated fruit, mainly for the 0.05 and 0.1 mM doses (Figure 2A,B). The chlorophyll content in fruit peel showed values of ca. 7.5 mg 100 g^−1^ at the first sampling date, without significant difference among treatments, and remained stable during the first two weeks of storage in the control and all treated fruit, whereas significant decreases (*p* < 0.05) occurred in the control and 0.01 mM melatonin-treated fruit from the second week of storage. This decreasing trend was observed after three weeks of storage in 0.05 and 0.1 mM melatonin-treated fruit, and it is worth noting that chlorophyll concentration at the last sampling date was significantly higher (*p* < 0.05) in these fruit (5.1–5.2 mg 100 g^−1^) than in the control and 0.1 mM melatonin-treated fruit with ca. 4.3 mg 100 g^−1^ (Figure 3A).

Weight loss increased during storage, although values were significantly lower (*p* < 0.05) in the 0.05 mM melatonin-treated fruit than in the control and the remaining melatonin-treated fruit (10.5–10.9%) until the third week of storage. However, at the last sampling date, weight loss reached values of 13–14%, without significant differences among the control and melatonin-treated fruit (data not shown). With respect to fruit firmness, significant decreases (*p* < 0.05) were observed during storage, from values of ≈35 N mm^−1^ at the first sampling date to 7.10 ± 1.51 N mm^−1^ in control fruit after 28 days of cold storage + 2 days at 20 °C. However, firmness levels were significantly higher (*p* < 0.05) in all melatonin-treated fruit than in the control after the first week of storage and during the whole storage period for the 0.05 mM dose (Figure 3B).

#### 2.2.2. Total Phenolic and Antioxidant Activity in Cherimoya Peel

**Figure 4:**
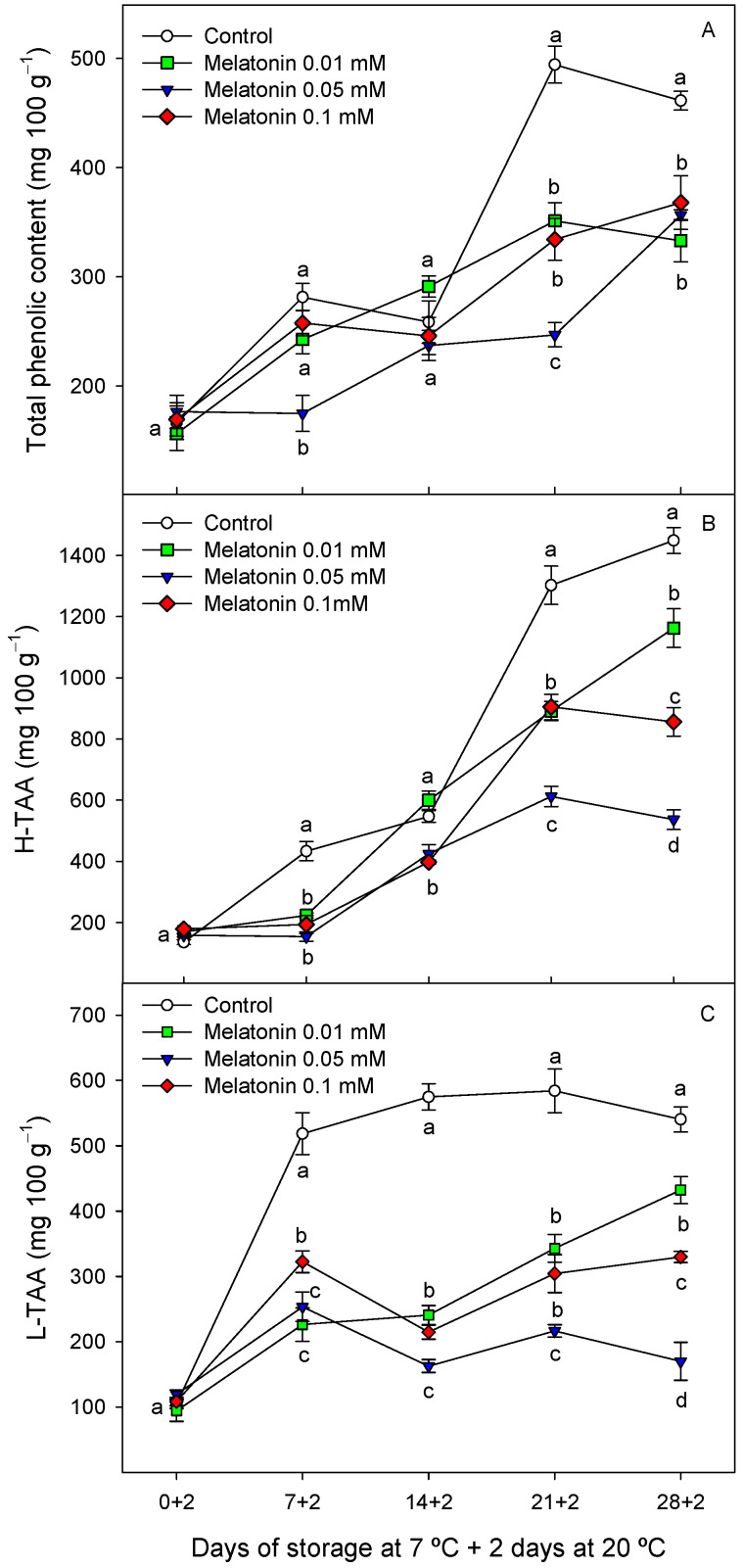
Total phenolic content (**A**), hydrophilic antioxidant activity (H-AA, (**B**)) and lipophilic antioxidant activity (L-AA, (**C**)) in cherimoya peel as affected by melatonin (0.01, 0.05 and 0.1 mM) treatments. Data are the mean ± SE of three replicates. Different letters show significant differences (*p* < 0.05) among treatments for each sampling date.

The total phenolic content in the peel of cherimoya fruit ranged from 156 to 176 mg 100 g^−1^ at the first sampling date and increased during storage, reaching final values of 461.26 ± 8.69 and ca. 350 mg 100 g^−1^ in the control and melatonin-treated fruit, respectively (Figure 4A). Similarly, hydrophilic antioxidant activity (H-TAA) increased during ripening, although the values were significantly higher (*p* < 0.05) in the control than in melatonin-treated fruit (Figure 4B). Lipophilic antioxidant activity (L-TAA) in the peel of control fruit increased sharply during the first week of storage, and thereafter remained almost stable. However, in the 0.01 and 0.1 mM melatonin-treated fruit, L-TAA in the peel increased during the whole storage period, whereas no changes occurred in the 0.05 mM-treated fruit (Figure 4C).

#### 2.2.3. Internal Fruit Quality Parameters

**Figure 5:**
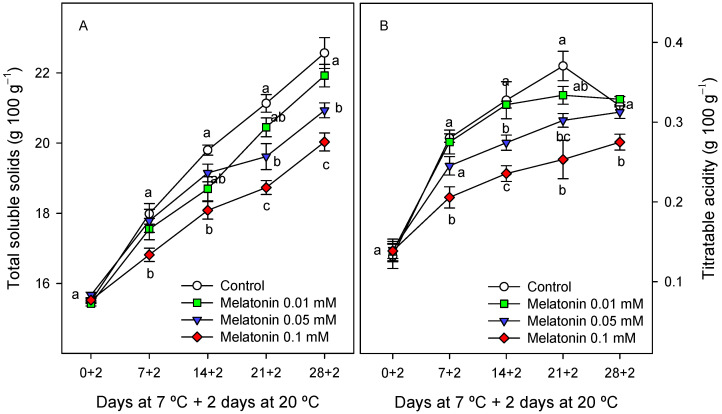
Total soluble solids (**A**) and titratable acidity (**B**) as affected by melatonin (0.01, 0.05 and 0.1 mM) treatments. Data are the mean ± SE of three replicates. Different letters show significant differences (*p* < 0.05) among treatments for each sampling date.

The total soluble solids (TSS) concentration in cherimoya pulp tissue at the first sampling date was similar in the control and melatonin-treated fruit, ca. 15.5 g 100 g^−1^, and a steady increase was observed during storage in the control and treated fruit, with values being, in general, significantly lower (*p* < 0.05) in melatonin-treated fruit than in the control, especially for 0.1 mM concentration (Figure 5A). In a similar way, increases were observed in titratable acidity (TA) from the first sampling date to the third week of storage, and then, no changes or slight decreases occurred. Nevertheless, it is worth mentioning that significantly higher (*p* < 0.05) values of TA were observed for control fruit with respect to melatonin-treated fruit during the whole storage time, these effects being dose-dependent (Figure 5B).

#### 2.2.4. Respiration Rate and Ethylene Production

**Figure 6:**
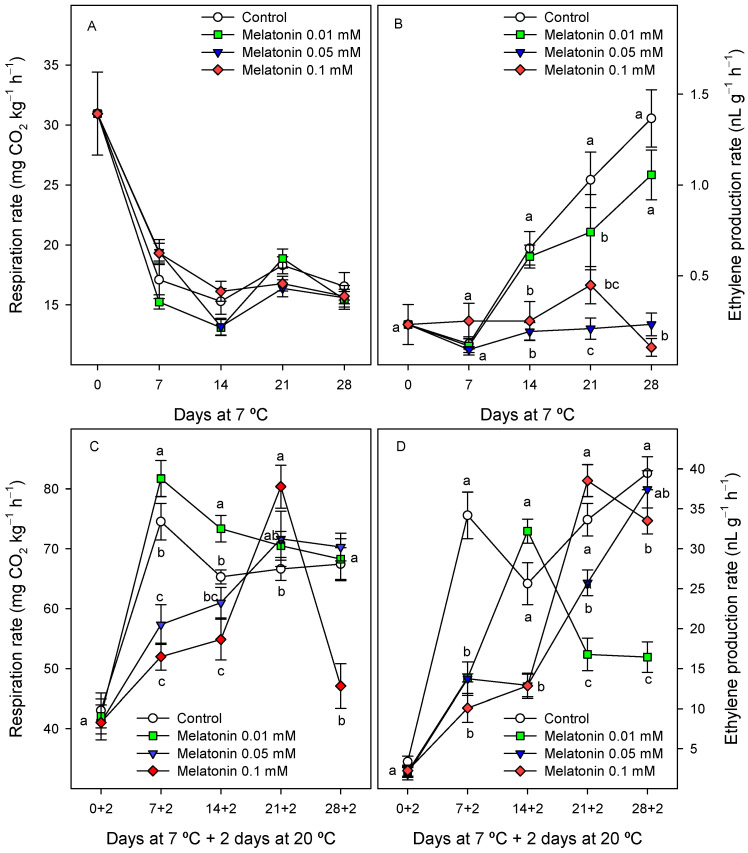
Respiration rate (**A**,**C**) and ethylene production (**B**,**D**) in control and 0.01, 0.05 and 0.1 mM melatonin-treated fruit during storage at 7 °C (**A**,**B**, for respiration and ethylene, respectively) and subsequent shelf life at 20 °C (**C**,**D**, for respiration and ethylene, respectively). Data are the mean ± SE of three replicates. Different letters show significant differences (*p* < 0.05) among treatments for each sampling date.

The respiration rate at harvest was 30.95 ± 3.46 mg CO_2_ kg^−1^ h^−1^ and decreased sharply to 16–19 mg CO_2_ kg^−1^ h^−1^ when cherimoya fruit were stored at 7 °C in both the control and in treated fruit, remaining stable during the whole cold storage period, without significant differences among treatments (Figure 6A). Ethylene production was very low in cherimoya fruit during the first week of storage at 7 °C, and increased thereafter in the control and 0.01 mM melatonin-treated fruit, whereas in the 0.1 and 0.05 mM treatments, the respiration rate remained at significantly lower levels (*p* < 0.05) until the end of storage (Figure 6B). However, the respiration rate and ethylene production increased in all cherimoya fruit when they were transferred for 2 days at 20 °C after cold storage. Considering the respiration rate, a peak was reached in the control and 0.01 mM melatonin-treated fruit after 7 days at 7 °C + 2 days at 20 °C, with values of ca. 75–80 mg CO_2_ kg^−1^ h^−1^, whereas this peak in respiration was reached after 21 days at 7 °C + 2 days at 20 °C in the 0.05 and 0.1 mM treatments (Figure 6C). Increases in ethylene production were also found when cherimoya fruit were transferred to 20 °C after cold storage. In the control fruit, a peak of 34.17 ± 2.91 nL g^−1^ h^−1^ was reached after 7 days at 7 °C + 2 days at 20 °C. A decrease then occurred from the second to the third week, and thereafter, an increasing trend was observed until the last sampling date. In melatonin-treated fruit, the peak in ethylene production was delayed for 1, 2 and 3 weeks at the 0.01, 0.05 and 0.1 mM melatonin concentrations, respectively, although the maxima values were similar and independent of the treatment, ranging from 33 to 38 nL g^−1^ h^−1^ (Figure 6D).

## 3. Discussion

Cherimoya fruit are generally harvested when the peel colour changes from dark green to light green or greenish-yellow, with L* values close to 60 and b* ca. 30–32 in the ‘Fino de Jete’ cultivar [29], which are similar to the values at harvest in the present experiments. CI symptoms in cherimoya fruit, manifested as peel browning, increased during storage, although they were significantly reduced by melatonin treatment, especially with 0.05 and 0.1 mM doses (Figure 1A), and the ion leakage in peel tissues was likewise reduced (Figure 1B). In fact, a high correlation was found between ion leakage and CI (y = 0.48x + 49; r^2^ = 0.723) when considering data of all cherimoya fruit and sampling dates during storage. The increase in ion leakage in CI-damaged fruit is due to an increased action of membrane lipid-degrading enzymes, leading to reductions in the concentration of unsaturated fatty acids and the unsaturated:saturated fatty acid ratio, increased cell membrane permeability and disruption of the compartmentalised function of the cell membranes [30,31,32]. Similar to the present results, melatonin postharvest treatments have been shown to increase chilling tolerance in several fruit species, such as litchi [31], pepper [32], guava [33], peach [34], longan [35] and banana fruit [36]. This effect has been attributed to the maintenance of cell membrane structure and permeability, due to a higher content of unsaturated fatty acids and enhanced antioxidant enzyme activities, which lead to lowering the accumulation of malondialdehyde (MDA) and reactive oxygen species (ROS) and maintaining the cellular redox state.

The browning process of cherimoya peel was manifested as decreases in L* and b* colour parameters, which decreased during the whole storage period in the control and 0.01 mM melatonin-treated fruit, but were maintained until the third week of storage in 0.05 and 0.1 mM melatonin-treated fruit (Figure 2A,B). The L* and b* colour parameters were negatively correlated with CI incidence: y = −0.09x + 51, r^2^ = 0.791; and y = −0.06x + 30.31, r^2^ = 0.661, respectively. In Figure S3, it can be observed that 0.05 mM melatonin-treated fruit had a visual quality optimum for consumption after 21 days of storage at 7 °C + 2 days at 20 °C, while the control and 0.01 and 0.1 mM melatonin-treated fruit were highly deteriorated. In addition, it is worth noting that after 28 days 7 °C + 2 days at 20 °C, all the fruit, both the control and treated fruit, showed severe browning symptoms and were unacceptable for consumption. These changes in peel colour could not be attributed to reduced chlorophyll concentration, which was maintained at levels similar to those at the first sampling date until the second week of storage in the control and treated fruit (Figure 3A), whereas significant colour changes were found in the control and 0.01 mM-treated fruit (Figure 2A,B). Accordingly, decreases in L* and b* colour parameters and visual colour changes were observed in the ‘Fino de Jete’ cultivar during 7 days of storage at 20 °C, without changes in peel chlorophyll concentration [6]. However, at the last sampling dates of cold storage, significant losses in peel chlorophyll concentration were observed, although they were delayed in fruit treated with 0.05 and 0.1 mM concentrations of melatonin, which could be attributed to the effect of these treatments on delaying senescence processes.

Cherimoya peel is rich in phenolic compounds compared with other fruit species, with 46 different phenolic compounds being identified recently, the major ones being quercetin derivatives, rutin and quinic acid [37,38]. However, there is no available literature about changes in peel phenolic content during storage of cherimoya fruit for comparative purposes. The present results showed an increasing trend in peel phenolic content during storage, as well as for H-TAA and L-TAA, which were delayed in melatonin-treated fruit compared with the control (Figure 4A–C). Similarly, melatonin treatment led to lower increases in total phenolic content in banana peel, which was associated with reduced CI symptoms [36], and similar results were observed in the flesh of ‘Friar’ plum [22]. In contrast, melatonin treatment reduced CI and increased phenolic and flavonoid content in four mango cultivars, due to higher activity of phenylalanine ammonia lyase and tyrosine ammonia lyase [23], as well as in longan pericarp [35] and in guava fruit [39], leading to enhanced antioxidant activity. Thus, the effects of melatonin treatment of phenolic content could be dependent on fruit tissue and fruit species.

Early reports used penetration tests to evaluate cherimoya softening [6,40]. However, the flesh of cherimoya fruit is composed of soft segments arranged around its longitudinal axis and contains many hard seeds; thus, the results of the penetration test may be highly biased by the presence of seeds and the segment orientation [40]. Thus, the compression test, as performed in the present experiments, is more suitable to measure cherimoya fruit firmness and is similar to the way that consumers subjectively estimate cherimoya fruit softness by applying a compression force to the fruit surface with the fingers. Cherimoya fruit soften quickly after harvest, the decreases in firmness being quicker at higher temperature and is attributed to cell wall hydrolytic enzymes, such as pectin methylesterase (PME), polygalacturonase (PG), xyloglucon endotransglycosylases and expansins [7,41,42,43]. This softening process was delayed by melatonin treatments, the largest delay of two weeks being found for the 0.05 mM concentration (Figure 3B). These effects could be due to a low activity of the cell wall hydrolytic enzymes and/or a delay in the expression of their coding genes, as reported for PG, PME and β-galactosidase activities in mango fruit as a consequence of melatonin treatment [26].

Cherimoya fruit is described as a climacteric fruit but has two particular peaks of respiration during ripening, the first one occurring 2–3 days after harvest and the second one after 7–10 days when stored at ambient temperature and with a peak in ethylene production after the first peak of respiration [6,8,10,11]. In addition, the time of the ethylene climacteric peak was independent of the harvest date [44] and reached different maxima values, up to 50–300 nL g^−1^ h^−1^, depending on the cultivar and storage temperature [7,41,42,45,46]. These previous papers also reported that changes related to organoleptic quality properties, such as softening and accumulation of sugars, organic acids and aroma compounds, started concomitantly with the increase in ethylene production, and the optimum quality for eating was reached at the ethylene peak. In the present experiment, melatonin treatments delayed the respiration rate and ethylene production peaks in a concentration-dependent manner, and also delayed the reduction in fruit firmness and the increases in TSS and TA. Similarly, 0.05 mM melatonin dipping treatments for 1 h of ‘Guifei’ mango delayed ethylene production and decreased 1-aminocyclopropane-1-carboxylic (ACC) content and the activities of ACC-synthase (ACS) and ACC-oxidase (ACO) [26]. Similar results were obtained in banana [27] and pear [28] fruit after melatonin dipping treatments at 0.05 mM for 2 h and 0.1 mM for 12 h, respectively, which were attributed to reduced expression of the genes coding for ACS and ACO enzymes. In contrast, Sun et al. [47] reported that melatonin dipping treatment at 0.05 mM for 2 h down-regulated the expression of genes coding for ACS and ACO in tomato fruit. Thus, it is clear that the effect of melatonin treatment on ethylene production and its physiological related process depends on the fruit species, applied dose and time of dipping treatment.

The increase in TSS during cherimoya ripening has been attributed to enhanced fructose and glucose concentrations, due to starch hydrolysis, an ethylene dependent process evolving more quickly with higher storage temperature [6,8,48]. With respect to TA, which usually decreases during postharvest fruit ripening [49], increases were found in cherimoya flesh during storage, according to previous reports, in ‘Fino de Jete’ and other cherimoya cultivars harvested at different dates and stored at different temperatures, due to enhanced concentrations of malic and citric acids [6,12,29]. However, the increase in TA was delayed in 0.05 and 0.1 mM melatonin-treated fruit with respect to controls. Taking together the results for ripening quality parameters, it could be inferred that melatonin delayed the cherimoya postharvest ripening process during storage, due to delayed ethylene production, as previously reported by other treatments, such as shock CO_2_ treatments for three days [12], 500 nL L^−1^ 1-methylcyclopropene (1-MCP) for 16 h [41] or an edible coating based on carnauba wax [50]. Furthermore, melatonin has been reported to delay senescence and ripening processes, leading to maintenance of fruit quality properties in guava [33], rambutan [51], strawberries [52] and peaches [34], among other fruit species. These effects have been attributed to a more efficient system for scavenging reactive oxygen species and reduced membrane lipid peroxidation. In addition, melatonin effects on delaying and/or reducing ethylene biosynthesis have been reported in climacteric fruit, such as mango [26], banana [27] and pear [28], which led to fruit quality maintenance.

Finally, it is worth noting that melatonin is commonly used for insomnia and improving sleep in different conditions, such as jet lag, depression, chronic pain or dementia, among others, in doses up to 8 mg a day for up to 6 months in adults. Melatonin is a dietary supplement approved for the U.S. Food and Drug Administration (US FDA), and there are no dosage restrictions for melatonin since it does not have side effects nor generate dependence [53]. In the present experiment, the best results were obtained with a 0.05 mM dose, which corresponds to 11.6 mg/L. Thus, even 1 L of melatonin solution would be safe for human consumption.

## 4. Materials and Methods

### 4.1. Plant Material and Melatonin Treatments

Experiment 1: Optimisation of melatonin concentration and time of dipping. Cherimoya (*Annona cherimola* Mill.) fruit of the “Fino de Jete” cultivar were manually harvested in a commercial plot located at Motril (Granada, Spain, Latitude: 36°45′02″ N Longitude: 3°31′04″ W). The fruit were harvested early in the morning from 12-year-old trees at the commercial ripening stage (16 November 2021) and immediately transferred to the laboratory in a in a refrigerated truck in 6 h. Fruit with uniform size and colour were randomly divided into 3 replicates of 75 fruit. Then, 15 lots of 5 fruit were allocated to each replicate for the following melatonin treatments: 0 (control), 0.1, 0.3, 0.5 and 1 mM by dipping for 10, 60 and 180 min. The experiment was replicated three times. Melatonin (Sigma-Aldrich, Darmstadt, Germany, purity > 98% M5250) solutions were freshly prepared in distilled water containing 0.05% Tween 20 before treatments. Then, fruit were left to dry at ambient temperature for 2 h and stored at 7 °C for 14 days plus 2 days at 20 °C. Thereafter, analytical determinations were carried out in each fruit (three replicates of five fruit).

Experiment 2: The best results in terms of quality maintenance of cherimoya fruit in the first experiment were obtained with 0.1 mM melatonin concentration and dipping for 10 min. Thus, for the second experiment, the treatment with 0.1 mM melatonin for 10 min was chosen and the other two lower concentrations, 0.05 and 0.01 mM applied for 10 min, were also assayed by using cherimoya fruit harvested on 11 January 2022 (as indicated in Experiment 1). The preparation of melatonin dipping solutions and the application of treatments were performed as previously indicated by using 3 replicates of 25 fruit per replicate for the control and each melatonin concentration (0.01, 0.05 and 0.1 mM). Then, cherimoya fruit were allowed to dry at room temperature, and thereafter, the fruit were stored at 7 °C to induce CI [10] for 0, 7, 14, 21 and 28 days. After each cold storage time, one lot of 5 fruit from each treatment and each one of the three replicates was selected at random and stored at 20 °C for 2 days before the following analytical determinations were performed.

### 4.2. Respiration Rate, Ethylene Production and Quality Parameters

The weight loss of individual cherimoya fruit was calculated by weighing the fruit before storage (initial weight) and after each sampling date (final weight) and was expressed as a percentage loss. The respiration rate and ethylene production were quantified in each individual fruit in duplicate by placing each fruit in a hermetic 1 L glass container for 1 h. After that, four samples of 1 mL of headspace atmosphere were taken by using airtight syringes. Two of these were injected into a gas chromatograph GC Shimadzu 14B (Shimadzu Europa GmbH, Duisburg, Germany) to measure CO_2_ concentration, and the other two were injected into a Shimadzu GC-2010 gas chromatograph (Shimadzu Europa GmbH, Duisburg, Germany) to measure ethylene concentration. The chromatographic conditions were previously described by Martínez-Romero et al. [54]. The respiration rate and ethylene production were expressed as mg of CO_2_ kg^−1^ h^−1^ and nL g^−1^ h^−1^, respectively. The colour parameters (L*, a* and b*) were individually measured in each fruit by using a Minolta colorimeter (CRC400, Minolta Camera Co.; Kantō, Tokio, Japan). Three readings along the fruit’s equatorial perimeter were made in each fruit. Fruit firmness was evaluated for each fruit by using a TA.XT-plus Texture Analyzer (Stable Mycrosystems, Godalming, UK) eqiped with a force that achieved a 5% deformation at the equator area on the fruit using a flat steel plate probe. The results were expressed as the force-deformation (N mm^−1^). External CI was assessed visually in each fruit and rated as the percentage of the superficial area showing browning. For all these parameters, the results are the mean ± SE of determinations performed individually in 5 fruit for each of the 3 replicates.

Electrolyte leakage (EL) was determined according to Mao et al. [55] with some modifications. From each treatment and replicate of five fruit, 15 peel discs (3 from each fruit) with 0.5 mm diameter were taken and rinsed 3 times for 3 min each with 50 mL of deionized water at room temperature with constant shaking. Then, discs were incubated in deionized water for 30 min and the electrical conductivity (EC) was measured (C1). Finally, the samples were boiled at 100 °C for 15 min and the electrical conductivity (C2) was measured again. EL was calculated using the following formula: EL = (C1/C2) 100, and the data, expressed as percentage, are the mean ± SE of 3 replicates.

For the chlorophyll measurements, one disc of 6.25 mm diameter of the peel tissue of each of the 5 fruit of each replicate were taken, weighed and placed immediately into 8 mL of 100% methanol. The chlorophyll extraction was left to occur in the dark at 30 °C for 24 h. The absorbance of the extracts was measured using a spectrophotometer (1900 UV/Vis, Shimadzu, Kyoto, Japan) at 652 and 665 nm [56], and the total chlorophyll concentration was expressed as mg 100 g^−1^ (mean ± SE).

A 10-g flesh sample of each of the 5 fruit of each replicate was taken and cut into small pieces to obtain a homogenous sample for each replicate. About 20 g of this sample was homogenised with distilled water (50:50 *w/v*) and centrifuged at 5000× *g* for 10 min. The TSS concentration was determined in duplicate in the supernatant with an Atago PR-101 digital refractometer and expressed as g 100 g^−1^ (mean ± SE). TA was determined, also in duplicate in each sample, by automatic titration (785 DMP Titrino, Metrohm, Herisau, Switzerland) and expressed as g of malic acid equivalent per 100 g^−1^ fresh weight (mean ± SE).

### 4.3. Total Phenolic Quantification and Total Antioxidant Activity

A longitudinal strip of peel tissue of the five fruit of each replicate was taken and ground with liquid N_2_ to obtain a homogeneous sample for each replicate, which was stored at −20 °C until further analysis of total phenolics and antioxidant activity. The total phenolics were measured by homogenising 2 g of frozen peel samples with 15 mL of water: methanol (2:8, *v/v*) containing 2.0 mM NaF. After centrifugation of the extracts at 10,000× *g* at 4 °C for 20 min, the total phenolic content (TPC) was measured in duplicate in each extract sample by using the Folin–Ciocalteau reagent, as previously reported [57], and the results (mean ± SE) were expressed as mg of gallic acid equivalent per 100 g^−1^ fresh weight. The total antioxidant activity was measured by using the ABTS test as described by Serna-Escolano et al. [57] with small modifications. In total, 10 mL of 50 mM phosphate buffer solution and 6 mL of ethyl acetate were added to 2 g of cherimoya peel sample, and the mixture was homogenized, as indicated for the phenolic extraction, and then centrifuged at 10,000× *g* for 20 min at 4 °C. The hydrophilic and lipophilic phases were separated, and the hydrophilic and lipophilic antioxidant activities (H-TAA and L-TAA) were measured in duplicate in each extract. The H-TAA determination was carried out with 890 μL of 50 mM glycine buffer solution mixed with 30 μL of 10 mM 2,2′-azinobis(3-ethylbenzothiazoline-6-sulfonic acid (ABTS) solution, 30 μL of 1 mM H_2_O_2_ and 25 μL of 10 µM peroxidase. The absorbance of this mixture was measured at 730 nm. Then, 25 μL of water-soluble phase extract was added to the preceding mixture, and the absorbance was measured again at 730 nm after 1 min. The results (mean ± SE) were expressed as mg of (±)-6-Hydroxy-2,5,7,8-tetramethylchromane-2-carboxylic acid (Trolox) equivalent per 100 g^−1^ of cherimoya peel with reference to the Trolox calibration curve. On the other hand, L-TAA determination was assayed with 30 μL of 10 mM ABTS solution mixed with 30 μL of 1 mM H_2_O_2_, 25 μL of 10 µm peroxidase and 850 μL of ethanol. The L-TAA was measured as above and the results (mean ± SE) were expressed in mg Trolox equivalent per 100 g^−1^ of peel weight with reference to the Trolox calibration curve.

### 4.4. Statistical Analysis

All data in this paper are expressed as mean ± standard error (SE) (n = 3). The data were subjected to analysis of variance (ANOVA). Mean comparisons were carried out using a multiple range test (Tukey’s HSD test) to find significant differences (*p* < 0.05). All the analyses were performed using the SPSS software package, version 22 (IBM Corp.; Armonk, NY, USA).

## 5. Conclusions

Our results show that melatonin treatments before storage at chilling temperatures could be a useful tool to decrease CI damage in cherimoya fruit, since ion leakage, browning and chlorophyll losses in skin tissues of treated fruit were delayed compared with the control. Moreover, additional effects of melatonin treatments on retarding postharvest ripening and senescence processes and on maintaining quality parameters were observed. According to the results of CI damage and quality parameters, the storage of cherimoya control fruit with optimal quality traits for consumption was 7 days at 7 °C + 2 days at 20 °C. Storage could be extended up to 14 days at 7 °C + 2 days at 20 °C for the 0.01 and 0.1 mM melatonin-treated fruit and up to 21 days at 7 °C + 2 days at 20 °C for the 0.05 mM dose. These effects were attributed to a delay in the climacteric ethylene production in treated fruit with respect to control. However, further research is needed in order to clarify the effect of melatonin on the activity and gene expression levels of the enzymes involved in ethylene biosynthesis.

## Data Availability

The data that support the findings of this study are available from the corresponding author upon request.

## Supplementary Materials

The following supporting information can be downloaded at: https://www.mdpi.com/article/10.3390/ijms24043787/s1.
