# Supplementary figures and images for: Melatonin Treatments Reduce Chilling Injury and Delay Ripening, Leading to Maintenance of Quality in Cherimoya Fruit
Source: Int J Mol Sci. 2023 Feb 14;24(4):3787. doi: 10.3390/ijms24043787 (PMC9960509; doi:10.3390/ijms24043787)

Supplementary Materials

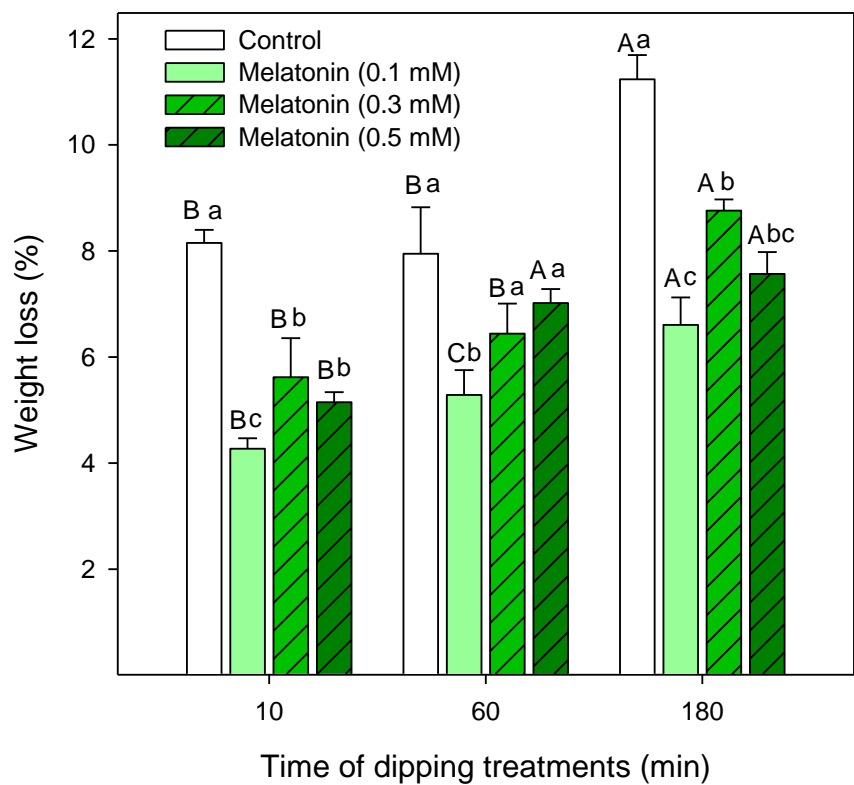

Figure S1

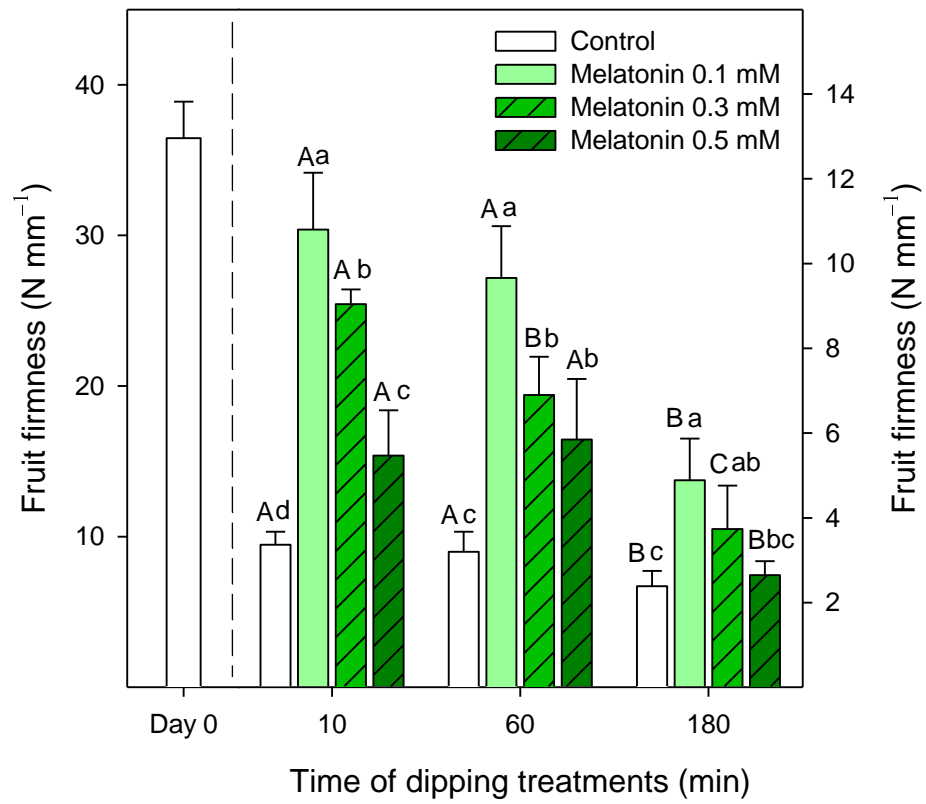

Figure S2

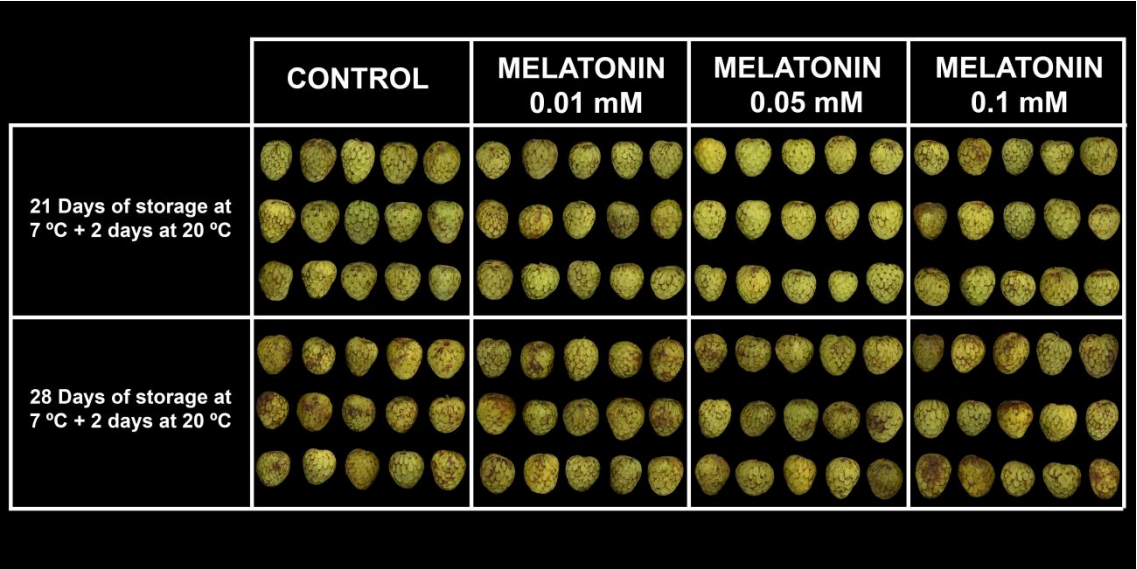

Figure S3

Supplement: Supplementary file 1 [file ijms-24-03787-s001.zip › ijms-2153244-supplementary.pdf]
